# Evolution of Energy Related Metabolites in Plasma from Newborns with Hypoxic-Ischemic Encephalopathy during Hypothermia Treatment

**DOI:** 10.1038/s41598-017-17202-7

**Published:** 2017-12-06

**Authors:** Ángel Sánchez-Illana, Antonio Núñez-Ramiro, María Cernada, Anna Parra-Llorca, Eva Valverde, Dorotea Blanco, Maria Teresa Moral-Pumarega, Fernando Cabañas, Hector Boix, Antonio Pavon, Mercedes Chaffanel, Isabel Benavente-Fernández, Inés Tofe, Begoña Loureiro, José R. Fernández-Lorenzo, Belén Fernández-Colomer, Ana García-Robles, Julia Kuligowski, Máximo Vento, HYPOTOP Study Group, Malaika Cordeiro, María Arriaga, Noelia Ureta-Velasco, M. Angeles Caballero, Cristina Fernández, Yolanda Castilla, J. F. Ferreira, Simón P. Lubián-López, Pilar Jaraba, Jon López de Heredia

**Affiliations:** 1 Neonatal Research Unit, Health Research Institute La Fe, Valencia, Avda. Fernando Abril Martorell 106, 46026 Valencia, Spain; 2 Hospital Universitario La Paz, Paseo de la Castellana 261, 28046 Madrid, Spain; 3 Hospital Universitario Gregorio Marañón, C/O’Donnell 48–50, 28009 Madrid, Spain; 4 Hospital Universitario 12 de Octubre, Avda. de Córdoba, s/n, 28041 Madrid, Spain; 5 Hospital Universitario Quirónsalud Madrid, Calle Diego de Velazquez s/n, 28223 Madrid, Spain; 6 Hospital Universitario Vall d’Hebron, Passeig Vall d’Hebron 119–129, 08035 Barcelona, Spain; 7 Hospital Universitario Virgen del Rocío, Avda. Manuel Siurot, s/n, 41013 Sevilla, Spain; 8 Hospital Materno Infantil Carlos Haya, Avda. Arroyo de los Angeles s/n, 29011 Málaga, Spain; 9 Hospital Universitario Puerta del Mar, Avda. Ana de Viya 21, 11009 Cádiz, Spain; 10 Hospital Universitario Reina Sofía, Avda. Menendez Pidal s/n, 14004 Córdoba, Spain; 11 Hospital Universitario Cruces, Plaza Cruces s/n, 48903 Barakaldo, Vizcaya Spain; 12 Hospital Álvaro Cunqueiro, Estrada Clara Campoamor 341, 36312 Vigo, Spain; 13 Hospital Central de Asturias, Avda. De Roma, 33011 Oviedo, Asturias Spain; 14 Division of Neonatology, University & Polytechnic Hospital La Fe, Avda. Fernando Abril Martorell 106, 46026 Valencia, Spain

## Abstract

Therapeutic hypothermia (TH) initiated within 6 h from birth is the most effective therapeutic approach for moderate to severe hypoxic-ischemic encephalopathy (HIE). However, underlying mechanisms and effects on the human metabolism are not yet fully understood. This work aims at studying the evolution of several energy related key metabolites in newborns with HIE undergoing TH employing gas chromatography – mass spectrometry. The method was validated following stringent FDA requirements and applied to 194 samples from a subgroup of newborns with HIE (N = 61) enrolled in a multicenter clinical trial (HYPOTOP) for the determination of lactate, pyruvate, ketone bodies and several Krebs cycle metabolites at different sampling time points. The analysis of plasma samples from newborns with HIE revealed a decrease of lactate, pyruvate and β-hydroxybutyrate concentrations, whereas rising malate concentrations were observed. In healthy control newborns (N = 19) significantly lower levels of pyruvate and lactate were found in comparison to age-matched newborns with HIE undergoing TH, whereas acetoacetate and β-hydroxybutyrate levels were clearly increased. Access to a validated analytical method and a controlled cohort of newborns with HIE undergoing hypothermia treatment for the first time allowed the in-depth study of the evolution of key metabolites of metabolic junctions in this special population.

## Introduction

Hypoxic-ischemic encephalopathy (HIE) secondary to perinatal asphyxia is a major cause of mortality and long-term neurologic co-morbidities especially occurring in the term neonate. Every year worldwide one million infants die and one million survive with neurological impairment. However, the overall incidence varies notably. Hence, while in developed countries the incidence of HIE ranges between 1 and 2 per 1000 live births, in low income areas it may account for 26 per 1000 live births^1^.

Therapeutic hypothermia (TH) initiated within 6 hours from birth^2^ was introduced to resuscitation guidelines in 2010^3^ and involves a core temperature reduction to 33.5 ± 0.5°C for 72 h. Since then hypothermia treatment is standard of care for infants with HIE. To date this is the only treatment that has shown to reduce death and long-term disability for infants especially with moderate encephalopathy. However, in severe cases the combined outcome of death and/or severe disability has not been as successful and still affects around 45% of babies^4^. Therefore, in order to improve outcome of severely affected neonates, new synergistic therapies are being explored^5,6^. To this end, the HYPOTOP trial (EudraCT #2011-005696-17), a randomized, multicenter, double blinded placebo-control trial, aiming at the evaluation of the neuroprotective effect of topiramate (TPM) in addition to moderate total body hypothermia in patients with HIE, has been carried out. The HYPOTOP trial aimed at reducing the hyperexcitability component of HIE which leads to increased neuronal apoptosis. TPM has rendered good results in this regard in previously launched pilot studies^7^. New anticonvulsant drugs such as TPM or levetiracetam have shown not to foster apoptosis and even inhibit cascades of damage activated after hypoxic-ischemic insults at paediatric doses, indicating that they potentially act as neuroprotectors in addition to their antiseizure effects^8^.

Both, early assessment of the severity of cerebral injury and the prediction of neurological outcomes are crucial for parental counselling, selection of the most appropriate early neuroprotective strategies and/or establishment of multidisciplinary interventions to lessen the severity of chronic morbidities. The diagnosis of an asphyxia process that evolves to HIE is based on prenatal clinical information (sentinel events), Apgar scores with special emphasis on neurological assessment of tone, response to stimuli or reflexes and cord blood gas analysis reflecting metabolic acidosis and increased lactate concentration. Amplitude-integrated electroencephalography (aEEG) in the first hours after birth and magnetic resonance imaging (MRI) of the brain and multichannel EEG (mchEEG) later on may further confirm the diagnosis^5^.

Little is known about the evolution of most biochemical markers in newborns with HIE during TH. The secondary phase of injury is characterized by a failure of oxidative metabolism, which is associated with exhaustion of ATP reserves leading to cytotoxic edema, hyperexcitability, cerebral reperfusion and ultimately, cell death by necrosis and/or apoptosis. Clinical neurodevelopmental outcomes at 1 and 4 years of age are closely correlated with the severity of the secondary failure of oxidative metabolism at 15 h after birth^9^. Hence, the classical biochemical evaluation of the severity of asphyxia in the first hours of life is based on serial determinations of arterial pH, base deficit (BD) and blood lactate^10^. In practice, they showed reasonable precision for correctly identifying absent versus severe HIE. However, due to its remarkable ability to adapt to profound and prolonged hypoxia, the healthy foetus frequently is capable of tolerating such insults even without evidence of injury. Hence, BD and lactate provide rather imprecise relationship with neonatal encephalopathy for the intermediate group^9^.

This study reports the evolution of eight metabolites including lactate, pyruvate, metabolites from the Krebs cycle and ketone bodies in plasma from newborns with HIE undergoing hypothermia treatment in comparison to a control group of samples collected from healthy term newborns. A gas chromatography – mass spectrometry (GC-MS) analytical method was developed and successfully validated, allowing the simultaneous quantification of the studied metabolites employing a sample volume of only 50 µL, thus enabling serial determinations from small volume blood samples. The availability of sound data from a controlled cohort of infants clears the way for studying the role of each metabolite in the clinical context of TH in newborns with HIE.

## Results

### Characteristics of the study population

Characteristics of the studied sub-population of the HYPOTOP trial as well as the control group are shown in Table 1. Between both groups, no significant differences were found for the gestational age, gender and birth weight. In the control group, the percentage of C-section was significantly lower. For all parameters used for the diagnosis of HIE in the delivery room (i.e. Apgar scores, cord pH, BE and lactate), highly significant differences were found as expected. Likewise, for treatments related to the resuscitation procedure (i.e. positive pressure ventilation, cardiac massage and use of O_2_ and adrenalin) significant differences between both studied populations were obtained.

**Table 1:** Patients’ characteristics.

Parameter	Control (N = 19)	HYPOTOP (N = 61)	p-value
Gestational Age (weeks, mean ± s)	38 ± 2	39 ± 2	>0.05
Gender (% male/female)	53/47	54/46	>0.05
Birth Weight (g ± s)	3200 ± 500	3300 ± 600	>0.05
Type of delivery (% vaginal/C-section)	79/21	44/56	<0.01
Apgar 1 (median (min–max))	10 (9–10)	1 (0–5)	<0.01
Apgar 5 (median (min–max))	10 (10–10)	3 (0–8)	<0.01
Cord pH (mean ± s)	7.31 ± 0.06	6.8 ± 1.0	<0.01
Cord BE (mEq L^−1^, mean ± s)	−2.00 ± 0.04	−16 ± 7	<0.01
Cord lactate (mmol L^−1^, mean ± s)	4.5 ± 1.6	14 ± 4	<0.01
Positive pressure ventilation (% Yes/No)	0/100	98/2	<0.01
O_2_>21% (% Yes/No)	0/100	97/3	<0.01
Cardiac Massage (% Yes/No)	0/100	56/44	<0.01
Adrenalin (% Yes/No)	0/100	49/51	<0.01

### Quantification of metabolites in plasma samples

Full scan spectra of individual standard solutions were recorded during method optimization. Based on the results (data not shown), selected ion monitoring (SIM) parameters listed in Table 2 were carefully chosen. Two m/z were recorded for each metabolite for quantification and confirmation.

**Table 2:** Data acquisition parameters and main figures of merit of the quantification method.

Analyte	IS	m/z Quantification	m/z Confirmation	RT ± s [min]	RI	Calibration Range [µM]	y = ax^2^ + bx + c			R^2^ ± s	SER	LLOD in Plasma [µM]	LLOQ in Plasma [µM]
							a ± s	b ± s	c ± s				
Pyruvate	Pyr-13C	174	158	6.00 ± 0.02	1056	0.8–100	—	1.02 ± 0.04	0.0111 ± 0.0016	0.9987 ± 0.0009	3	0.11	0.3
Pyr-13C	—	175	—	6.00 ± 0.02	—	—	—	—	—	—	—	—	—
Lactate	—	191	117	6.20 ± 0.02	1068	3.9–1000	—	12000 ± 2000	90000 ± 20000	0.994 ± 0.007	0.3	0.5	1.6
Acetoacetate	DMBA	188	89	7.260 ± 0.003	1141	3.1–100	0.011 ± 0.003	0.018 ± 0.008	−0.0007 ± 0.0005	0.9961 ± 0.0012	0.003	0.4	1.3
β-hydroxybutyrate	DMBA	191	117	7.640 ± 0.008	1167	0.4–100	—	0.4 ± 0.3	−0.0001 ± 0.0008	0.9983 ± 0.0009	0.7	0.06	0.2
Succinate	DMBA	247	147	9.770 ± 0.002	1319	0.1–25	0.17 ± 0.06	0.308 ± 0.014	0.00090 ± 0.00003	0.9965 ± 0.0017	0.08	0.014	0.04
Fumarate	DMBA	245	147	10.200 ± 0.002	1351	0.1–25	—	1.4 ± 0.7	0.0002 ± 0.0002	0.994 ± 0.005	0.00007	0.014	0.04
Malate	DMBA	147	233	12.130 ± 0.002	1501	0.1–25	—	1.18 ± 0.02	0 ± 0	0.994 ± 0.006	0.02	0.014	0.04
α-ketoglutarate	DMBA	198	204	13.20 ± 0.05	1588	0.1–25	0.1536 ± 0.0003	0.19 ± 0.04	0 ± 0	0.996 ± 0.004	0.015	0.014	0.04
DMBA	—	239	—	14.700 ± 0.004	—	—	—	—	—	—	—	—	—

Note: RT, Standard Error of Residuals (SER) measured on Day 1; LLOQs were established as the concentration of analyte that can be measured with an imprecision of less than 20% and a deviation from target of less than 20% and taking into account the preconcentration factor of 2.4 achieved during sample processing. The LLOQ is defined as three times the LOD. RI stands for Retention Index calculated as $RI=100\times n+100\times ({t}_{c}-{t}_{n})/({t}_{n+1}-{t}_{n})$, where $c$ stands for compound of interest, $n$ stands for alkane with $n$ carbon atoms eluting before compound $\,c$ and $n+1$ stands for alkane with $n+1\,\,$carbon atoms eluting after compound c. ${t}_{c}$,$\,{t}_{n}$ and ${t}_{n+1}$ represent their respective retention times.

After completing method optimization, analytical figures of merit were assessed during method validation including precision, selectivity, lower limit of detection (LLOD), lower limit of quantification (LLOQ), sample dilution, carry-over as well as sample and standard stabilities. Method validation was carried out following the recommendations of the US Food and Drug Administration (FDA) guidelines for bioanalytical method validation^11^. However, since the FDA guideline aims at the quantitative analysis of drugs and their metabolites it cannot be directly applied for the measurement of endogenous metabolites due to the lack of blank matrices. This drawback has been circumvented applying recovery tests of spiked plasma samples.

Table 2 summarizes the characteristics of the obtained calibration lines for each studied metabolite. When possible, linear regression was employed for calibration lines. However, for acetoacetate, succinate and α-ketoglutarate the use of a second order polynomial fit was necessary in order to cover a sufficiently wide concentration range. With the exception of lactate, signal normalization employing an IS was necessary. All calibration curves had a coefficient of determination (R^2^) of ≥0.990.

**Figure 1:**
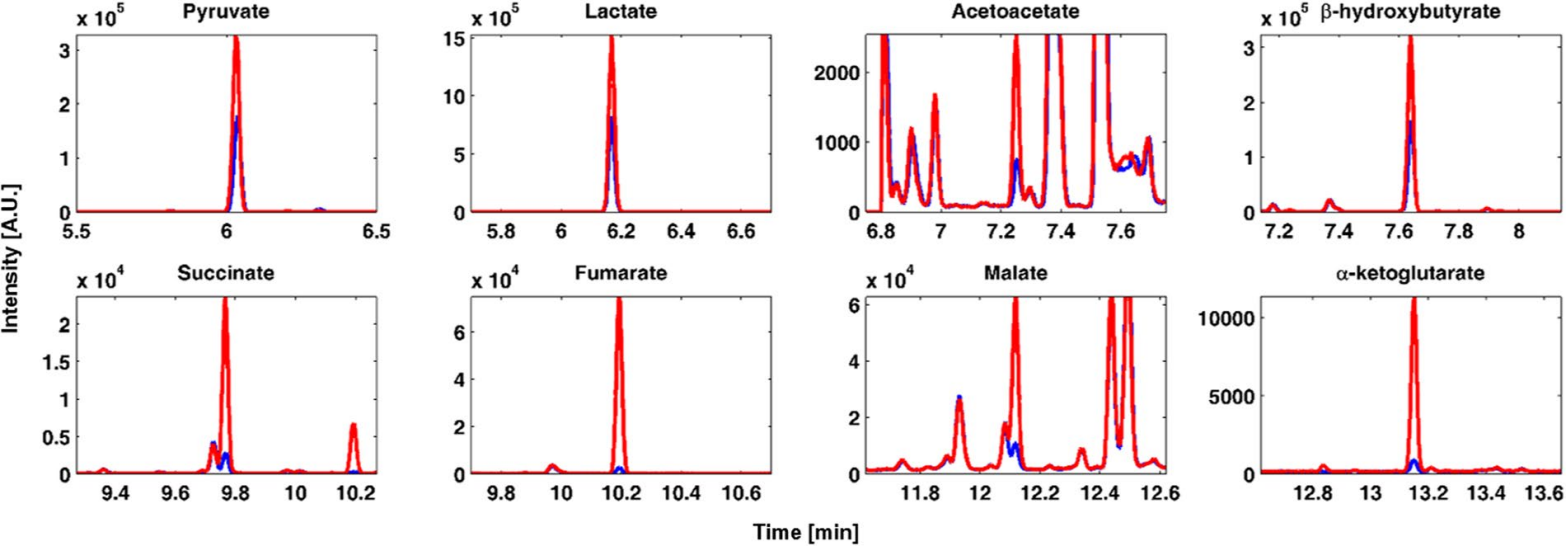
Chromatograms acquired during the injection of a plasma sample (blue line) and a spiked plasma sample (red line). Note: for lactate the 1:10 diluted sample is depicted.

Figure 1 shows SIM chromatograms obtained during the analysis of a representative sample before and after spiking. It can be observed that in this plasma sample, all studied metabolites were detected with the exception of fumarate. Chromatographic resolution >1 from other unknown matrix compounds was achieved for all metabolites with the exception of malate. High retention time stability (see Table 2), combined with the use of two characteristic m/z was used to assure the specificity of the signal. In addition, the Retention Index reported in Table 2 was determined for each metabolite and results fitted well with values reported in literature^12^. LLOQs listed in Table 2 were established as the concentration of analyte that can be measured with an imprecision of less than 20% and a deviation from target of less than 20% and taking into account the preconcentration factor of 2.4 achieved during sample processing. The LLOQ was defined as three times the LLOD.

Table 3 shows intra- and inter-day accuracy and precision levels given as % recoveries ± standard deviations (s) obtained for standard solutions as well as spiked samples at low, medium and high concentration levels. For standard solutions, imprecision determined at each concentration level did not exceed 15%, except for the LLOQ, where it did not exceed 20%, while at the same time a deviation from the target concentration of less than 15% (20% at the LLOQ) was achieved. Results from the recovery study carried out with spiked plasma samples revealed the magnitude of matrix effect for each studied metabolite. No significant matrix effect was observed for pyruvate, succinate, malate and α-ketoglutarate. In case of the remaining metabolites, recoveries were ranging in a ±30% interval for precision as well as accuracy.

**Table 3:** Back-calculated accuracy and precision of standard solutions and plasma sample at three spiking levels.

Analyte	Standard solutions - % Accuracy ± s (conc µmol L^−1^)						Spiked plasma samples - % Accuracy ± s (conc µmol L^−1^)					
	Intra-day (N = 3)			Inter-day (N = 3)			Intra-day (N = 3)			Inter-day (N = 3)		
	Low	Medium	High	Low	Medium	High	Low	Medium	High	Low	Medium	High
Pyruvate	94 ± 20 (0.8)	109 ± 1 (25)	96.8 ± 0.1 (100)	90 ± 6 (0.8)	105 ± 4 (25)	96 ± 3 (100)	92 ± 10 (20)	92 ± 4 (40)	85.0 ± 1.0 (50)	94 ± 7 (20)	97 ± 5 (40)	89 ± 3 (50)
Lactate	115 ± 18 (3.9)	93 ± 7 (250)	97 ± 11 (1000)	114 ± 3 (3.9)	96 ± 3 (250)	99 ± 4 (1000)	69 ± 10 (250)	96 ± 14 (50)*	95 ± 5 (100)*	83 ± 14 (250)	120 ± 30 (50)*	100 ± 30 (100)*
Acetoacetate	108 ± 7 (3.1)	96 ± 4 (25)	100.9 ± 1.8 (100)	107.3 ± 0.5 (3.1)	102 ± 8 (25)	100.2 ± 1.6 (100)	70 ± 50 (10)	72 ± 18 (20)	69 ± 7 (50)	90 ± 20 (10)	68 ± 18 (20)	71 ± 14 (50)
β-hydroxybutyrate	112.6 ± 1.8 (0.4)	103 ± 6 (25)	97.3 ± 3.8 (100)	103 ± 9 (0.4)	104 ± 3 (25)	97.8 ± 0.4 (100)	65 ± 17 (10)	69 ± 3 (20)	63 ± 3 (50)	77 ± 12 (10)	80 ± 30 (20)	74 ± 13 (50)
Succinate	119 ± 12 (0.1)	100 ± 2 (6.3)	97.9 ± 0.8 (25)	118 ± 9 (0.1)	102 ± 4 (6.3)	97.2 ± 0.6 (25)	85 ± 10 (2)	93 ± 7 (4)	87.0 ± 0.8 (10)	99 ± 16 (2)	110 ± 20 (4)	95 ± 11 (10)
Fumarate	96 ± 2 (0.1)	87 ± 2 (6.3)	105.9 ± 0.9 (25)	103 ± 6 (0.1)	95 ± 7 (6.3)	102 ± 3 (25)	74 ± 8 (2)	80 ± 6 (4)	62.00 ± 0.17 (10)	80 ± 30 (2)	100 ± 30 (4)	80 ± 30 (10)
Malate	94 ± 3 (0.1)	87 ± 2 (6.3)	104.5 ± 0.7 (25)	109 ± 13 (0.1)	94 ± 7 (6.3)	103 ± 3 (25)	82 ± 11 (2)	87 ± 7 (4)	97.0 ± 0.5 (10)	94 ± 17 (2)	100 ± 30 (4)	103 ± 17 (10)
α-ketoglutarate	93 ± 16 (0.1)	105 ± 3 (6.3)	96.5 ± 1.1 (25)	100 ± 7 (0.1)	107.6 ± 1.7 (6.3)	99 ± 2 (25)	97 ± 11 (2)	103 ± 9 (4)	98.0 ± 0.9 (10)	111 ± 12 (2)	111 ± 10 (4)	114 ± 17 (10)

Note: Values within brackets indicate the concentration of each metabolite in the standard solution/spiking concentration.

*Measured in 1:10 diluted sample.

The stability of the studied metabolites in standards and samples was assayed under different conditions and compared to freshly prepared standards and samples. After three cycles of freeze-thawing, concentrations found in standards and samples remained unchanged with recoveries ranging between 81 and 120 and 80 and 114%, respectively. After 24 h at 4 °C, which are typical autosampler conditions, metabolites were stable in standards and plasma samples with recoveries of 86 to 112 and 93 to 115%, respectively, indicating that injection sequences of up to 24 h will not alter the obtained results. No alteration of the measured concentrations was observed (recoveries between 86 and 113%) from the analysis of individual aqueous standard solutions stored during 30 days at −20 °C. Analysis of samples collected and stored at −80 °C for at least two months did not show significant changes in determined concentrations (N = 5, p > 0.05), indicating that sample storage under the assayed conditions was appropriate, facilitating the application of the method in clinical trials as well as research studies.

### Survey of clinical samples

**Figure 2:**
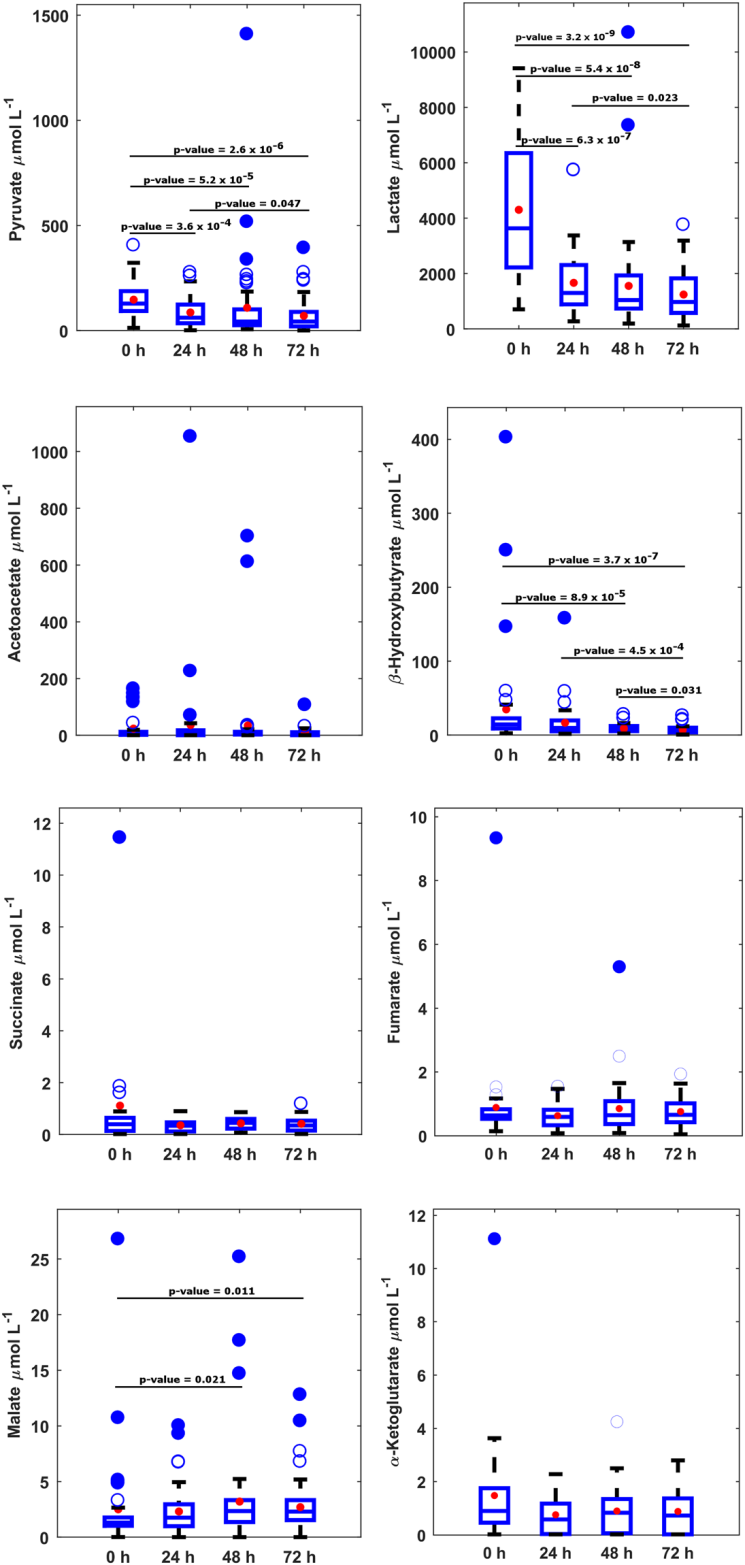
Boxplots of metabolite concentrations in plasma samples from newborns included in the HYPOTOP trial. Note: acetoacetate, succinate, malate and α-ketoglutarate detected in 77, 95, 92 and 60% and other analytes in 100% of samples, concentrations < LLOQ were set to 1/2xLLOQ; p-values calculated employing the two-tailed Wilcoxon rank sum test for equal medians.

The validated GC-MS method was applied for the analysis of 194 plasma samples drawn after birth at different points in time from newborns enrolled in the HYPOTOP trial. Figure 2 represents metabolite concentrations at different sampling time points. Whereas some metabolites (i.e. acetoacetate, succinate, fumarate and α-ketoglutarate) remained constant throughout the first three days of life, others showed significant changes (two-tailed Wilcoxon rank sum test for equal medians, α = 0.05, p-value for each metabolite shown in Fig. 2) with time. Accordingly, lactate, pyruvate and β-hydroxybutyrate concentrations decreased, whereas rising malate concentrations were observed. When comparing metabolite concentrations of newborns treated with TPM or placebo stratified by sampling time points, no significant differences were observed at 24 h, 48 h or 72 h after the administration of the first dose (data not shown).

Furthermore, plasma samples from healthy, term newborns were collected and analysed for the sake of comparison. Main descriptors of the distribution of plasma metabolite concentrations of the control group in comparison to newborns subjected to hypothermia treatment 48 h after the administration of the first dose of TPM are summarized in Table 4. Significantly lower levels of pyruvate and lactate were found in healthy control newborns, whereas acetoacetate and β-hydroxybutyrate levels were clearly increased (two-tailed Wilcoxon rank sum test for equal medians, α = 0.05, p-value for each metabolite shown in Table 4).

**Table 4:** Main descriptors of the distribution of concentrations (µmol L^−1^) in plasma samples collected from the control group (N = 19) and newborns enrolled in the HYPOTOP trial 48 h after the administration of the first dose of TPM. Note: p-value calculated employing the two-tailed Wilcoxon rank sum test for equal medians (α = 0.05).

Metabolite	Control Group				HYPOTOP (48 h)				p-value
	Range P_10_-P_90_	Median	Mean ± s	>LLOQ (%)	Range P_10_-P_90_	Median	Mean ± s	>LLOQ (%)	
Pyruvate	6–50	11	100 ± 200	100	12–200	40	100 ± 200	100	1.6 × 10^−5^
Lactate	300–1000	700	700 ± 400	100	500–3000	1000	1600 ± 2000	100	0.002
Acetoacetate	20–700	100	300 ± 300	100	0.7–30	7	35 ± 130	82	1.7 × 10^−8^
β-Hydroxybutyrate	13–700	150	300 ± 300	100	4–15	8	9 ± 5	100	4.9 × 10^−8^
Succinate	0.13–0.7	0.3	0.4 ± 0.3	95	0.11–0.8	0.5	0.4 ± 0.2	100	0.235
Fumarate	0.5–1.1	0.7	0.7 ± 0.2	79	0.3–1.3	0.6	0.8 ± 0.8	100	0.234
Malate	1.0–8	1.2	3 ± 4	100	0.7–5	2	3 ± 4	92	0.535
α-Ketoglutarate	0.5–2	1.2	1.3 ± 0.6	74	0.02–7	1.2	2 ± 4	81	0.672

## Discussion

**Figure 3:**
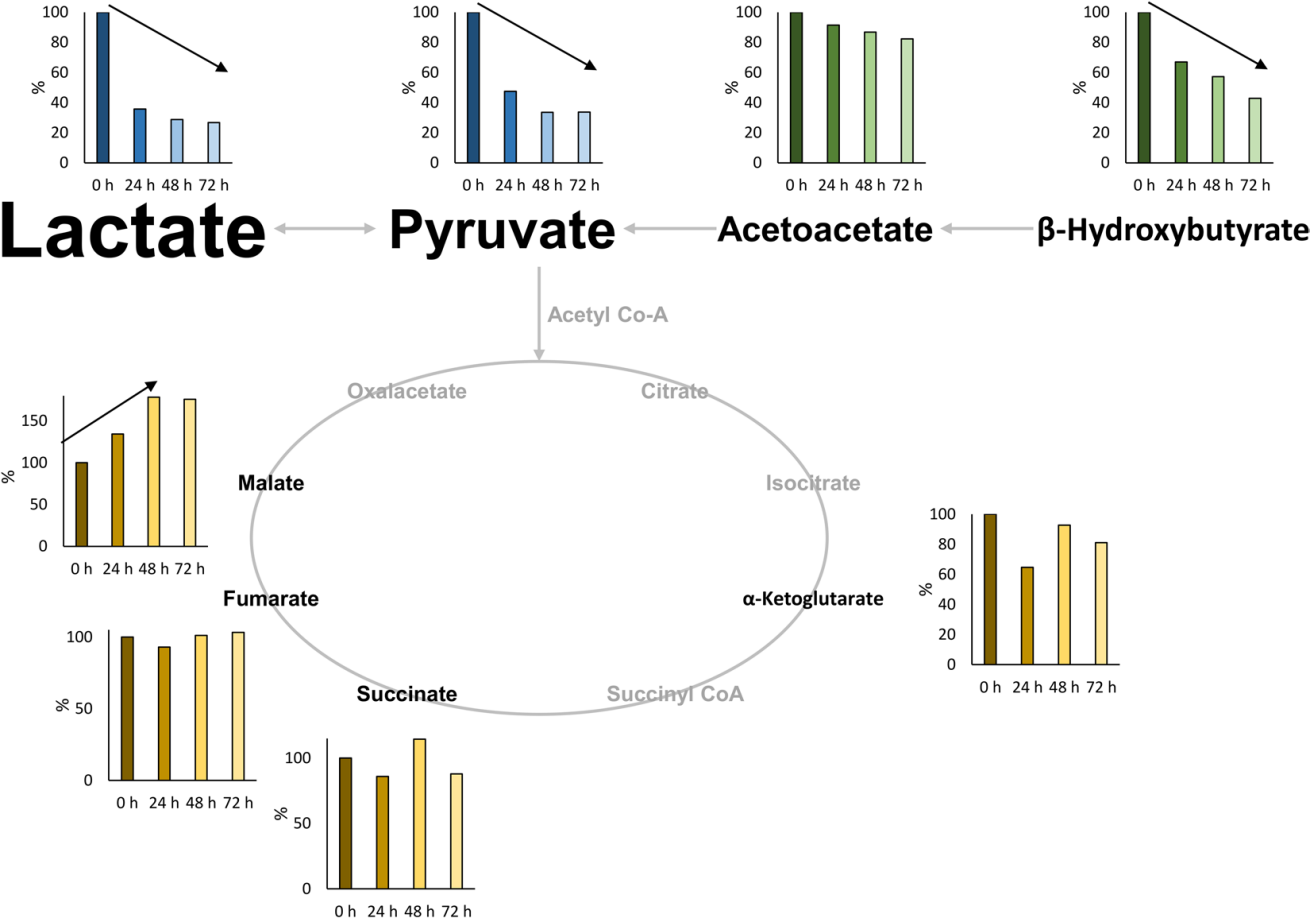
Relative changes of metabolites as a function of age of newborns with HIE enrolled in the HYPOTOP trial. Note: median values as a relative measure to median values at t0; letter size proportional to concentration levels; arrows indicate tendencies.

Evidence linking systemic aerobic metabolism and neurological disease in perinatal asphyxia and subsequent HIE has been reported in animal models^13,14^ and in clinical studies^15,16^. Undoubtedly, whole body moderate hypothermia has substantially increased sequel free survival of newborn infants with moderate to severe HIE. A key beneficial physiologic effect of TH after perinatal asphyxia is the associated reduction in cerebral and whole-body metabolic rates by 5–8% for every 1 °C reduction of core temperature. Yet, a decrease in corporal temperature does not simply provoke a slowdown of the metabolism, but alters diverse functions of many macromolecules simultaneously, including enzyme activities and transport efficiency, suggesting that cooling leads to coordinated effects on multiple regulatory processes^17^. It can therefore be considered to be of utmost importance to study the evolution of key metabolites of metabolic junctions in newborns with HIE undergoing TH in order to gain a better understanding of the physiological response of these babies for optimizing clinical monitoring and treatment. This work aimed at shedding light into the evolution of several key metabolites related to energy metabolism, including lactate, together with pyruvate, the ketone bodies acetoacetate and β-hydroxybutyrate as well as several Krebs cycle intermediates, in newborns with HIE receiving TH during the first 72 hours of life (see Fig. 3).

In recent years, several techniques have emerged that allow covering several of those metabolites in a single analysis in biological samples providing appropriate levels of sensitivity, accuracy and precision. In the literature, mainly GC-MS based methods have been reported^18–21^. Alternatively, nuclear magnetic resonance^16^, liquid chromatography with fluorescence^22^ or MS detection^23,24^ or capillary electrophoresis-MS^25,26^ have been employed. Here, a GC-MS based approach was developed for the quantification of metabolites in plasma samples from newborns. The validation study proved that the achieved levels of accuracy and precision were adequate for the simultaneous quantification of the set of metabolites in a sample volume of 50 µL.

Concerns about stability of ketone bodies and lactate in plasma samples have been raised and some authors propose the use of stabilizing agents for storage^27–29^. In this study metabolite stability in standards and samples was assessed and no significant alterations of concentrations were detected under the tested conditions. Hence, it can be concluded that the addition of stabilizing agents was not necessary and helps simplifying the sample collection process.

Under aerobic conditions glucose is metabolized through the glycolytic pathway and penetrates in the mitochondria where pyruvate is converted to acetyl coenzyme A, enters the tricarboxylic acid cycle and couples to the respiratory chain yielding energy in the form of adenosine triphosphate through oxidative phosphorylation^30^. In capillary blood drawn on day 4 from healthy full-term newborns, lactate and pyruvate were reported to range between 367 and 3245 and 10 and 141 μmol L^−1^, respectively^31^. In this study, for healthy term newborns at two days of age ranges (percentile 10 to 90) between 300 and 1000 and 6 and 50 μmol L^−1^ (see Table 4) were obtained for lactate and pyruvate, respectively. During hypoxia-ischemia decreased cerebral perfusion reduces the delivery of oxygen and glucose to the brain. Oxidative phosphorylation is blocked and pyruvate is converted into L-lactate through the anaerobic metabolism^5,32^. Anaerobic metabolism is by far less energy efficient than aerobic metabolism leading to energy exhaustion in brain cells. Initial lactate levels are a useful biochemical marker to assess the degree of birth asphyxia^9,32,33^. It has been demonstrated that lactate levels took longer to normalize in asphyxiated newborns with moderate to severe neonatal encephalopathy compared with newborns with mild neonatal encephalopathy^34^. In a retrospective case series Balushi *et al*.^32^ suggest that lactate levels during the first 4 days of life should be carefully monitored in asphyxiated term newborns treated with hypothermia in order to optimize handling of those patients and alleviate brain injury. Furthermore, during hypothermia peripheral perfusion is significantly decreased and lactate production may indicate hypoxia at tissue level even in the absence of metabolic acidosis^35^. This study for the first time reports ranges and evolution of lactate values in newborns with HIE during TH. This knowledge is important for the interpretation of lactate values of babies during TH monitored in the neonatal intensive care unit. In the cohort studied in this clinical trial an increase in lactate levels was found in the acute stage and a decreasing tendency with time was observed as expected, revealing a partial restoring of aerobic metabolism upon clinical stabilization as a consequence of the energy saving mechanisms triggered by TH.

Pyruvate showed increased levels during the whole examined time period and concentrations also decreased with time although remaining elevated as compared to the control group at 48 h of life (see Fig. 2). In rat brain tissue, the activation of the anaerobic flux during hypoxia-ischemia and increased glycolysis has been reported with all rate-limiting enzymes activated, being the transport of glucose across the blood-brain barrier the major rate-limiting step of this process. Consequently, the authors found increased pyruvate and lactate levels in brain during hypoxia-ischemia^36^. Here, reported results corroborate the accumulation of pyruvate during hypoxia-ischemia and show the evolution of its concentration profile during TH.

Whereas in adults glucose is essentially the sole energy fuel for the brain, in neonates the ketone bodies acetoacetate and β-hydroxybutyrate derived from ketogenesis in the liver are likely to be as important^17,27,37^. Furthermore ketones have been found to act as neuroprotectors^38^. In neonates suffering from severe HIE depletion of ketones has been reported suggesting that systemic metabolic responses such as ketogenesis may play a key role in preventing neurological injury during asphyxia^16^. Here, significantly higher levels of both metabolites were found in newborns from the control group presumably due to the high fat content of mother’s milk consumed by healthy babies. In newborns with HIE, both metabolites showed a decrease with time (although not statistically significant for acetoacetate), which might probably be attributed to their consumption as energy fuels. A recent study^17^ revealed that, hypothermia achieves its neuroprotective effects by mediating the cellular acetylation status through a coordinated suppression of acetyl-CoA, a metabolite that resides in metabolic junctions of glycolysis, amino-acid catabolism and ketosis. Both pyruvate and ketone bodies are major sources for acetyl-CoA, and were found to decrease under hypothermia conditions in rat brain.

Succinate accumulation has been associated with severe HIE, possibly evidencing HIF-1α mediated neurological injury^16^. Chouchanie *et al*. showed that the malate/aspartate shuttle and purine nucleotide cycle pathways increase fumarate production, which is then converted to succinate by succinate dehydrogenase reversal. The selective accumulation of succinate has been described as a universal metabolic signature of ischemia in a range of mouse and rat tissues and is thought to be responsible for mitochondrial ROS production that initiate ischemia-reperfusion injury^39^.

The evolution of intermediates of the Krebs cycle has been studied in the HYPOTOP cohort. Determined levels of succinate, fumarate, malate, and α-ketoglutarate did not show statistically significant alterations in comparison to normal control babies. Malate showed an increasing tendency with time, although levels found at 48 h were not different from those found in the control group.

The HYPOTOP trial aimed at the assessment of the neuroprotective effects of the administration of TPM as compared to a placebo. Results presented in this study regarding metabolites involved in central metabolic pathways did not reveal any effect of the administration of TPM on metabolite levels. Ongoing studies will focus on the evaluation of the effect of TPM on other metabolic pathways as well as imaging (MRI) and short-and long-term clinical outcomes of the babies enrolled in the HYPOTOP trial.

This study has some limitations. Hypothermia for HIE has become a universally accepted standard of care. Therefore, energy metabolism derived metabolites in asphyxiated patients without cooling could not be studied and compared to patients undergoing hypothermia treatment. Moreover, patients are recruited shortly after birth in a severe clinical condition and submitted to acute resuscitation manoeuvres, and cooled within 6 h after birth. Intriguingly, we did not find changes in energy-linked metabolites of the tricarboxylic cycle. Remarkably, most experimental models lack an active intervention to overcome asphyxia-derived damage. However, in our study human newborns were treated with hypothermia, sedation, analgesia and energy supplies in the form of parenteral nutrition to overcome the negative consequences of HIE. Altogether these interventions attenuated the rate of ATP consumption and enhanced its synthesis during the secondary energy failure phase (5, 6, 7). This allowed a satisfactory recovery of most organs of the body such as heart, kidney, intestine, muscle, and liver, while brain recovery depended on the initial degree of brain damage during primary energy failure. Determinations were carried out in plasma samples reflecting whole body dynamics and not specifically the brain. However, the value of the present study relates to the possibility of studying a big number of patients exquisitely controlled in a randomized controlled trial and opens a very valuable window for future studies.

In conclusion, this work presents data from a validated analytical approach for the determination of eight metabolites in small volume plasma samples. Furthermore, it reports for the first time the evolution of metabolite levels of newborns suffering from HIE with time during TH. The findings were discussed in the context of previously reported studies in animals and humans shedding light into the effect of TH on metabolite levels in newborns with HIE.

## Materials and Methods

### Standards and reagents

Sodium pyruvate, sodium lactate, lithium acetoacetate, sodium β-hydroxybutyrate, disodium succinate, sodium fumarate dibasic, malic acid and α-ketoglutaric acid potassium salt with purities >98%, as well as heparin sodium salt, methoxyamine hydrochloride (98%), pyrimidine (≥98%) and N-methyl-N-(trimethylsilyl)trifluoroacetamide with 1% trimethylchlorosilane (MSTFA + 1% TMCS), pyruvate-^13^C (Pyr^13^C) (99%) and 3,4-dimetoxibenzoic acid (DMBA) (99%) were purchased from Sigma-Aldrich Química SL (Madrid, Spain). Pyr^13^C and DMBA were used as internal standards (IS). Mixture of C_7_–C_40_ n-alkanes, each at 1000 g L^−1^ were acquired from Sigma-Aldrich Química SL (Madrid, Spain). Acetonitrile (analytical grade) and hexane (analytical grade) were obtained from J.T. Baker (Center Valley, USA) and Scharlau (Barcelona, Spain), respectively. Ultrapure H_2_O was generated with a Milli-Q purification system from Merck Millipore (Darmstadt, Germany).

### Population

**Figure 4:**
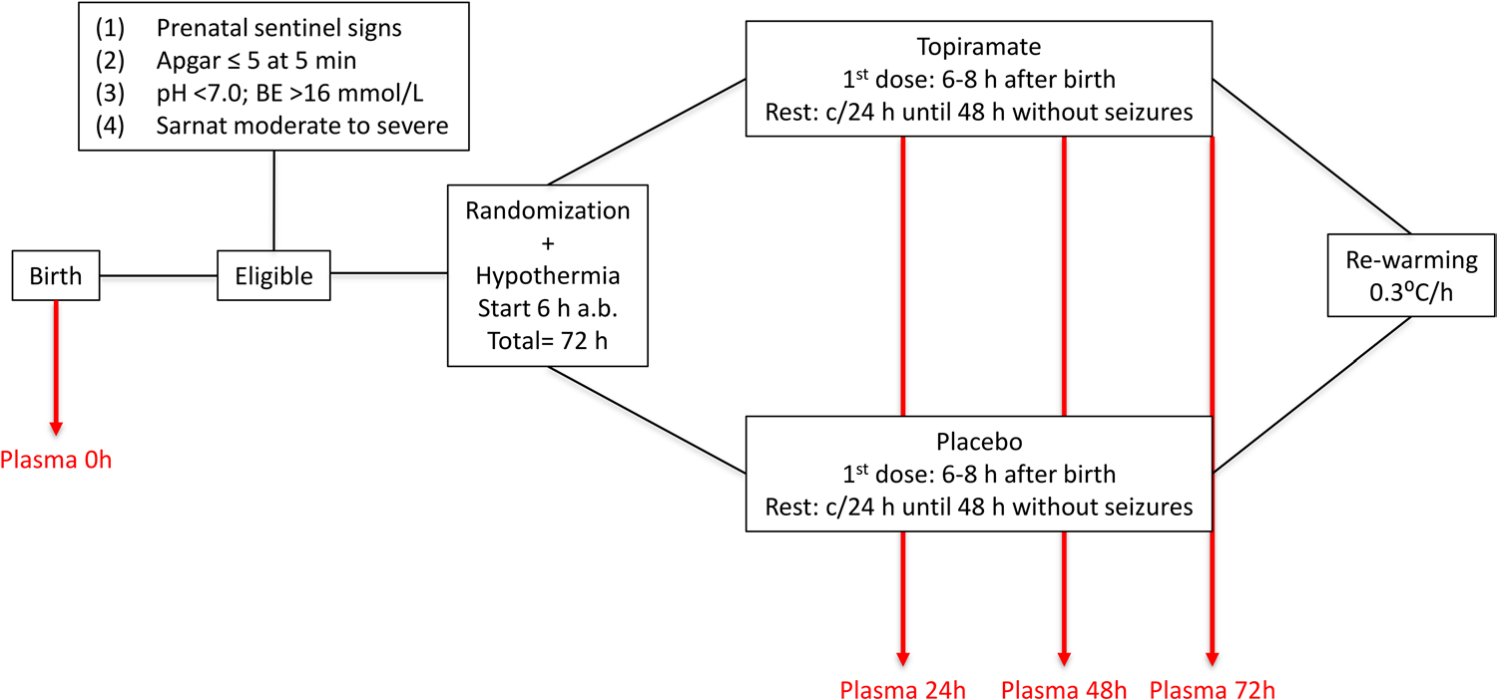
Flow diagram of the HYPOTOP trial.

A subgroup of newborns (N = 61) enrolled in the HYPOTOP trial (EU Clinical Trials Register: EudraCT 2011-005696-17, start date: 2013-06-18) was included in this study. The flow diagram presented in Fig. 4 summarizes the study protocol. Eligible patients were infants >36 weeks’ gestational age that fulfilled the following criteria: (1) prenatal signs compatible with hypoxia-ischemia such as alterations of foetal cardiac monitoring, abnormal foetal scalp pH (<7.2) or sentinel events such as *abruptio* placenta, meconium stained amniotic fluid or cord prolapse; (2) objective assessment of postnatal depression which included: Apgar ≤5 at 5 min, need for resuscitation with positive pressure ventilation for >10 min after birth, cord pH ≤ 7.0 and BE ≥−16 mEq L^−1^ in the worst blood gases obtained in the first 60 min after birth; (3) moderate to severe neurological status according to a modified Sarnat & Sarnat scale^40^. Exclusion criteria included gestational age < 36 weeks, birth weight < 2500 g, severe congenital malformations, chromosomopathies, or moribund status.

A total of 194 plasma samples were withdrawn at birth (0 h, corresponding to umbilical cord blood or, if not available, the first extracted blood sample, N = 41) and 24 h (N = 51), 48 h (N = 51) and 72 h (N = 51) after the administration of the first dose of TPM or placebo. For plasma collection, 0.5 mL of blood was drawn by venipuncture employing a heparinized syringe (1% sodium heparin). Plasma was obtained immediately thereafter to avoid sample degradation, centrifuging the samples at 1800 g during 10 min at 20°C. Supernatants were immediately collected and stored at −80 °C until analysis. As a control group (N = 19), excess volumes from blood samples extracted 53 ± 13 h after birth for routine neonatal screening from healthy term newborns before hospital discharge were employed and plasma was extracted following the same protocol as described above. Patient characteristics are summarized in Table 1. The Ethics Committee for Biomedical Research of the Health Research Institute La Fe (Valencia, Spain) approved the study protocol. Informed consent was obtained from parents of all participants. All methods were performed in accordance with the relevant guidelines and regulations.

### Preparation of stock, working and standard solutions

5 mL of individual stock solutions of pyruvate, lactate, acetoacetate, β-hydroxybutyrate, succinate, fumarate, malate and α-ketoglutarate at a concentration of 1 mmol L^−1^ were prepared in H_2_O by weighing of pure solid analytical standards in volumetric flasks. 1 mL aliquots of each standard solution were stored at −20 °C in order to avoid freeze-thawing of standard solutions. 500 µL of a working solution containing 1000 µmol L^−1^ of lactate, 100 µmol L^−1^ of pyruvate, acetoacetate and β-hydroxybutyrate and 25 µmol L^−1^ of succinate, fumarate, malate and α-ketoglutarate was prepared in H_2_O:CH_3_CN (1:4 v/v). 10 standard solutions were obtained by serial dilution from the working solution with H_2_O: CH_3_CN (1:4 v/v). Concentrations were ranging between 3.9 and 1000 µmol L^−1^ for lactate, 0.4 and 100 µmol L^−1^ for pyruvate, acetoacetate and β-hydroxybutyrate and 0.1 and 25 µmol L^−1^ for succinate, fumarate, malate and α-ketoglutarate. On each of the three validation days, 500 µL of spiking solution was prepared in H_2_O:CH_3_CN (1:4, v/v) containing 10 mmol L^−1^ of lactate, 800 µmol L^−1^ of pyruvate, 400 µmol L^−1^ of acetoacetate and β-hydroxybutyrate and 80 µmol L^−1^ of succinate, fumarate, malate and α-ketoglutarate.

### Biomarker analysis of plasma samples

Prior to analysis, standards and samples were derivatised in a two-step oximation-silylation procedure. Blanks were prepared in the same way as plasma samples, replacing the plasma volume with H_2_O.

Plasma samples were thawed on ice and homogenized on a vortex mixer during 30 s. 250 µL of cold (4 °C) CH_3_CN were added to 50 µL of plasma. Samples were maintained on ice during 5 min followed by centrifugation at 11000 g during 10 min at 4 °C. 200 µL of supernatant or standard solution were transferred to an Eppendorf® tube and 8 and 4 µL of Pyr^13^C and DMBA, both at a concentration of 1 mM were added, respectively. For the recovery test, spiked samples at three concentration levels (i.e. low, medium and high) were prepared by adding 2.5, 5 or 10 µL of spiking solution in addition to the IS. Samples and standards were evaporated on a SpeedVac concentrator from Genevac Ltd (Ipswich, UK) at 40 °C. Dry residues were suspended in 20 µL of a freshly prepared 4% (w/w) methoxyamine solution in pyridine. Samples and standards were incubated during 90 min at 30 °C on a thermomixer (MKR 13, Ditabis) under agitation. Then, 20 µL MSTFA + 1% TMCS were added. After 30 min of reaction time at 37 °C under agitation, samples and standards were diluted with 40 µL of hexane and placed in capped glass vials for GC-MS analysis. Samples were re-analysed after 1:10 dilution with hexane in case analyte concentrations exceed the established quantification range.

A 6890GC-5973N gas chromatography electron impact quadrupole mass spectrometric (GC-(EI)-Q-MS) system equipped with an autosampler and a HP-5MS column (0.25 mm × 30 m, film thickness 0.25 µm (5% Phenyl)-methylpolysiloxane) from Agilent Technologies (Santa Clara, CA, USA) were employed for sample analysis. The GC was operated at a constant He carrier gas flow at a flow rate of 1.2 mL min^−1^. The injector temperature was set to 260 °C and 1 µL of sample was injected at a split ratio of 5:1. The oven temperature was maintained at 60 °C during 1 min followed by a linear gradient of 10 °C min^−1^ until reaching 310 °C which were held during 10 min. The total runtime was 36 min.

### Data Availability

The datasets generated during the current study are available from the corresponding author on reasonable request.
